# Linkage to TB care: A qualitative study to understand linkage from the patients’ perspective in the Western Cape Province, South Africa

**DOI:** 10.1371/journal.pone.0260200

**Published:** 2021-11-19

**Authors:** Nosivuyile Vanqa, Graeme Hoddinott, Baxolele Mbenyana, Muhammad Osman, Sue-Ann Meehan

**Affiliations:** Department of Paediatrics and Child Health, Desmond Tutu TB Centre, Stellenbosch University, Tygerberg, South Africa; South African Medical Research Council, SOUTH AFRICA

## Abstract

**Background:**

Delayed linkage to tuberculosis (TB) treatment leads to poor patient outcomes and increased onward transmission. Between 12% and 25% of people diagnosed with TB are never linked to a primary health care facility for continued care. The TB health program is for creating processes that promote and facilitates easy access to care. We explored how TB patients experience TB services and how this influenced their choices around linkage to TB care and treatment.

**Methods:**

We enrolled 20 participants routinely diagnosed with TB in hospital or at primary health care facilities (PHC) in a high TB/HIV burdened peri-urban community in South Africa. Using the Western Cape Provincial Health Data centre (PHDC) which consolidates person-level clinical data, we used dates of diagnosis and treatment initiation to select participants who had been linked (immediately, after a delay, or never). Between June 2019 and January 2020, we facilitated in-depth discussions to explore both the participants’ experience of their TB diagnosis and their journey around linking to TB care at a primary health care facility. We analysed the data using case descriptions.

**Results:**

Twelve of twenty (12/20) participants interviewed who experienced a delay linking were diagnosed at the hospital. Participants who experienced delays in linking or never linked explained this as a result of lack of information and support from health care providers. Unpleasant previous TB treatment episodes made it difficult to ‘face’ TB again and being uncertain of their TB diagnosis. In contrast, participants said the main motivator for linking was a personal will to get better.

**Conclusion:**

The health care system, especially in hospitals, should focus on strengthening patient-centred care. Communication and clear messaging on TB processes is key, to prepare patients in transitioning from a hospital setting to PHC facilities for continuation of care. This should not just include a thorough explanation of their TB diagnosis but ensure that patients understand treatment processes. Former TB patients may require additional counselling and support to re-engage in care.

## Background

South Africa is the 8^th^ highest tuberculosis (TB) burdened country in the world and has the second highest TB incidence rate of 610/100 000 [1]. TB treatment coverage is 58% [1], highlighting a major gap between the estimated incidence and those treated for TB. The gap between diagnosis and TB treatment initiation at TB treatment facilities, known as initial loss to follow up (ILTFU), is between 12% and 25% [2–4]. Patients, who have engaged with health services and received a TB diagnosis, but are never linked to a TB treatment facility for continued care, is a gap in the TB program. Understanding this gap from the patients’ perspective is vital prior to designing interventions to close this gap.

ILTFU is a wide-spread problem in high TB burden countries [5], and will continue to exist unless efforts are made to assist patients with linkage. Patients not successfully linked to primary health care facilities (PHC) risk disease progression [6], continue to transmit TB [7], and become an overall cost driver for the health system [8].

Linkage to TB treatment is complex, with multiple health system challenges intersecting with patient health-seeking behaviour. Health system challenges include the data recording and management system [9], long waiting periods and negative health worker attitudes towards patients [10]. In 2014, a study using qualitative methods was conducted in five different provinces in South Africa [6]. Two groups were purposively selected; those accessing treatment and those categorised as ILTFU. They found that TB stigma, an association of TB with HIV, consuming alcohol, smoking, and difficulty accessing health facilities were the main barriers to linkage to care. Some studies suggest that delayed linkage to TB care is also related to patient factors and suggesting sub-optimal health-seeking behaviour [6, 11], while others highlight health system failures [5, 12]. Currently, there are no empirical data available from people who experience delays in linking to TB care. In the context of this study “linkage to care” is defined as being registered and on treatment at the same or different TB facility following a TB diagnosis. We aimed to explore TB patients’ experiences of linkage to care.

## Methods

### Study design

This exploratory study with qualitative data was a sub-study within a larger demonstration study (LINKEDin), which aimed to reduce ILTFU among TB patients in South Africa. This exploratory study aimed to understand patient experiences of linking to TB care. We also aimed to examine this in relation to whether the patient linked soon after diagnosis (without any follow up from the health services), experienced a delay in linkage (after follow up by health services) or not linked to care.

### Setting

This study was implemented in the Khayelitsha sub-district of the City of Cape Town District, Western Cape Province (WCP) of South Africa. Khayelitsha is a peri-urban low resourced area with a mix of formal and densely populated informal housing. It is one of the high TB burdened areas within the Western Cape Province.

TB services in Khayelitsha are provided at thirteen PHC facilities and one district hospital. The TB program is nurse-driven, providing TB testing and treatment of both drug susceptible and drug-resistant TB services at the PHC facility level. Patients are registered in a paper-based or electronic TB treatment register at facilities. All paper-based registers are captured into the electronic register, which is managed at the sub-district level and integrated at the district level.

Hospitals are not TB registration facilities in WCP. TB patients are registered when they access care at a PHC facility or a TB hospital for follow-up TB care and treatment. TB patients who have positive TB results following testing at either hospital or at a PHC facility but do not return for treatment initiation and registration are followed up routinely telephonically and by a community health worker (CHW) through a home visit.

### Study population and sampling

Unique to the WCP is the Provincial Health Data centre (PHDC), which consolidates person-level clinical data across government services to support patient care [13]. The PHDC integrates multiple routine health data sources (e.g. laboratory, pharmacy, TB registers) into single patient records, enabling health workers to monitor and easily manage patients across health facilities in the province [13].

The parent LINKEDin study used the PHDC to identify newly diagnosed TB patients and those patients who had not linked to a TB treatment facility. An automated step-by-step intervention was implemented; SMS messages were sent to patients not linked within three days of their diagnosis, telephone calls were made to patients not linked within three days after the SMS was sent and lastly, referrals to community care workers to do home visits were made for patients not linked within three days of the telephone call.

Within this sub-study, we used the PHDC to identify all adult (18-years and older) TB patients who had been diagnosed between March and September 2019 in the sub-district. We used the follow up data recorded in the PHDC from the LINKEDin study to purposively sample potential participants from four mutually exclusive categories of patients: i) those linked to a PHC facility almost immediately after diagnosis without any follow ups, ii) those linked after a short message service (SMS) or telephone call (experienced a delay), iii) those linked after being referred to a CHW for a home visit (experienced a delay) and iv) those who had no evidence within the PHDC of having been linked to a PHC facility.

Within each category we purposively selected patients for diversity, including where they were diagnosed (hospital or PHC facility), age and sex. We continued this process until we had at least 20 potential participants per category. We excluded those who did not have usable contact details or physical addresses recorded in the PHDC. We were left with 25 potential participants (between 5 and 7) per category. We contacted all 25 potential participants to explain the study and invite them to participate, 2 refused to participate, 2 were deceased and 1 reported being too ill to participate. We enrolled 20 participants (5 per category), by visiting them in their homes to obtain written informed consent.

### Data collection processes

We conducted in-depth discussions (approximately 2–4 discussion sessions per participant, with a total n = 47 discussion sessions of approximately ~45-60mins each) between June 2019 and January 2020. The initial plan was to conduct one or two in-depth discussions per participant, but we allowed the participants to guide the process. As the discussions continued, the participants wanted to share more information and so required more time and therefore we increased the number of discussion sessions per participant. The intervals between discussions varied between participants to, i) give more time and flexibility to share their narratives, and ii) accommodate participant availability. Discussion sessions were conducted either inside the participants’ home or in a motor vehicle for improved privacy, depending on their comfort and at their request.

Data were collected by two graduate researchers (NV and BM) who have experience and training in qualitative methods and interview skills. They designed the discussion guide, with oversight provided by a senior socio-behavioural scientist (GH). The researchers had practice sessions to familiarise themselves with the interview questions and prepared by discussing different scenarios to determine how best to approach sensitive topics that may arise. During data collection, the researchers worked together using a discussion guide, which allowed flexibility and iteration between topics. The topics covered *(a) kinship*, *(b) the patients’ TB history and illness narratives*, *(c) perceptions about community understanding and narratives around TB*, *(d) suggestions and recommendations of participants on how and what could be improved on the TB services*. All discussions were digitally recorded. The researchers took notes during all discussions, and wrote detailed reflections on the participants’ responses after each session. Both researchers shared a first language with the participants and were local to the city. This enabled them to understand the participants’ local references and explanations of their experiences relative to local context. Further, NV is a professional nurse with experience working in the local health system facilitating her interpretation of the participants’ descriptions of their journeys through the local health system.

### Analysis

We conducted a case descriptive analysis Ayres, Kavanaugh & Knafl [14]; Baskarada [15]. The analysis started with debriefing sessions between the researchers immediately after having facilitated each discussion in which we made notes about the context in which statements were made by participants. This was followed by a reflective case description (script) for each participants’ experiences from the series of discussions, guided by topic areas in the discussion guide. Both researchers listened to the audio recordings multiple times whilst creating the case descriptions to ensure accuracy to the participants’ narratives [16]. We then synthesized across the two researcher’s case descriptions for each participant. Direct quotes were transcribed when the researchers felt these illustrated points made in the case description. We then compared case descriptions, first within categories of research participants and then between all four linkage categories.

This process guided the selection of key main themes from data. These themes were selected to highlight the main findings across categories.

### Ethical considerations

The Health Research Ethics Committee of Stellenbosch University (N18/07/069) approved the study, which was conducted according to the guiding principles within the Declaration of Helsinki. Approvals were also received from the Western Cape Department of Health (NHRD ref: WC_201808_034) and the City of Cape Town Health Directorate. All participants were literate and able to understand the purpose of the study. They were all given an opportunity to ask questions and signed written informed consent prior the commencement of interviews. Participation was completely voluntary, and the participants were able to withdraw from the study at any time. This was explained at all interview sessions. Personal identifiers were removed from transcripts before the analysis and the text reported here uses pseudonyms.

## Results

Twenty participants; 11 men and 9 women were included in the study. The majority were diagnosed in hospital (12/20; 60%). Half (50%) were in the age category 35–44 and 2 (10%) were younger than 25-years. A third (6/20; 30%) had drug-resistant TB and 13/20 (65%) were co-infected with HIV. Almost half (8/20; 40%) had experienced at least one previous TB disease episode. We identified four key factors that influenced participants’ linkage to a PHC facility for TB care and treatment, *i) Lack of information from health workers*, *ii) Previous negative experiences*, *iii) Uncertainty regarding the TB diagnosis*, *and iv) Motivation to reduce impact of illness on self and others*.

### Lack of information from health workers

Most participants were generally well informed about TB symptoms–they described drenching night sweats, excessive cough, and unexplained weight loss. They were also aware of the different types of TB and treatment periods. However, many reported that they were not provided with clear information about the outcome of their diagnosis or the urgent need to access a PHC facility. This was consistent with their past TB disease episode for those who had more than one TB episode. Communication gaps were often about when and where they should go to get their TB test results. Some of the participants with linkage delays explained that they did not know that they had TB–that is, they had not had their TB test result communicated to them. These were mainly patients who tested at the hospital and were discharged before receiving their TB results. One participant who had been tested for TB whilst at the hospital had not received any further information from the staff about what to expect next. His reaction to receiving a text message informing him to visit the local PHC facility was:

*“I saw the message and did not understand so I ignored it because I was waiting for my appointment date, you know they shout at you when you don’t follow your dates” (man, 61-years-old).*

Similarly, another participant had been told by hospital staff that she would need to visit a PHC facility to initiate treatment, but felt disempowered to do so because she did not have an official result from the hospital to hand to the PHC facility:

*“I wasn’t given any letter at the hospital but they said I must go to the clinic. What was I going to say when I get there because there is nothing written about what is wrong with me? And you know how those people are in that TB room [referring to clinic staff] so I stayed at home and kept on going back to the private doctor until someone came to my house” (woman, 41-years-old).*

These examples illustrate that the participants did not clearly understand what steps they needed to take in order to access TB care. They explained that healthcare workers failed to adequately communicate to them what the next step of their journey to TB care should be and provide them with the information needed to take these steps. Overall, participants most often described receiving information that they should go to a PHC facility but much less detail on *how* or *why*. The net result was that these clients lacked confidence in their ability to insist on follow-up care at the PHC facility and waited on further in-person instruction at a subsequent health service interaction. This subsequent interaction was often not linked to TB but rather when accessing other chronic care services.

One participant reported having received results with ‘no TB detected’ at her initial TB test. However, there were further diagnostics performed that she was unaware of and these confirmed that she had TB:

*“When I was at the hospital they said I didn’t have TB so I stayed at home and waited for my next appointment but this thing kept growing [referring to a growth in her neck] so I came to stay with my cousin. No wonder they could not find me there (woman, 40-years-old)”*

### Previous negative experiences

Many participants who delayed linkage to TB care had experienced TB previously. These previous treatment experiences had been unpleasant. One man who completely ignored his results said:

*“I knew it was TB from the beginning, but I could not bring myself to be injected every day and take those horrible tablets” (man, 42-years-old).*

Another participant decided to ignore his diagnosis and not inform any family members to avoid being asked about TB treatment:

*“I won’t lie to you and say I had a reason not to go to the clinic I didn’t, I just don’t like the clinic that’s what happens every time I have TB, I stop the treatment because I get tired of it” (man, 30-years-old).*

A woman who had had multiple previous TB episodes decided to ignore her current TB diagnosis because she had had TB many times in the past:

*“I have so many illnesses and taking medication for HIV, and I didn’t go there for TB I broke my leg, I know how TB feels like, when they told me about TB I didn’t care. I already had TB 6 times remember, so what must I do?” (woman, 60-years-old).*

Similarly, another participant who was being treated for the second episode of drug-resistant TB said:

*“This is the 4^th^ time having TB and I don’t like these drugs, they make me nauseous, sometimes skip taking them because I don’t feel like it [taking the medication]” (woman, 37-years-old).*

Apart from physical ailments and side effects of TB treatment, the actions of the nursing staff during the previous TB disease episode influenced some participants’ decisions around seeking care again. One participant explained that she had previously been treated badly by healthcare staff:

*“Sometimes those people at the clinic can be very rude [referring to the clinic staff], I had doubts about going there in the first place, and guess what? One nurse in the weighing room asked me ‘why did I have TB’–accusing me that I defaulted on my ARVs and I was very hurt because how am I supposed to know why or how I got TB” (woman, 41-years-old).*

One young man had had a bad experience at the health facility; he was asked to come back on another day. This resulted in him never returning, he explained:

*“I went back to the clinic and told them I received a call to come and start TB treatment, they just told me to come back on a different day without any further explanation after all the efforts I made. That is why I never went back” (man, 36-years-old).*

### Uncertainty regarding the TB diagnosis

The majority of participants who were aware of their TB diagnosis but did not have the typical TB symptoms were reluctant to start treatment. When probed about this decision, they expressed that they strongly believed that they might have another condition that the nurses/doctors had mistaken for TB.

*“This doesn’t make sense, I have too many illnesses and maybe these doctors get confused themselves and also because I get this TB all the time. Why is it every time I don’t feel anything? so they might think it’s TB while it’s something else, I think it’s complicated and I just don’t get a doctor who will know exactly what I have and treat it for good” (woman, 60years-old).*

Similarly, participants who had different views and beliefs about what was wrong with them, took a decision to delay or not access TB care at all.

#### Alternative explanations for symptoms from traditional health beliefs

For some, not believing the diagnosis was linked with beliefs about an underlying supernatural cause of disease. When probed about this reaction one participant explained:

*“You know a lot of things happen in our lives [referring to African traditional beliefs that diseases have an underlying cause in disharmony with kin and community] and this is the 5^th^ time I get TB and I am not coughing again, why? Just like the last time they said I had MDR TB and I did not take those pills I threw them away and never told anyone, but I was cured, how? (woman, 37-years-old).*

Similarly, the example below is from a young man who believed that there are people in his community who are jealous of him/his family and therefore might have a hand in him/his family becoming ill.

*“Remember what I told you about my cousin who died after they said it was TB and this was shortly after people from this street were saying things and jealous of her progress in life. Again, now it’s me, how? Because I don’t smoke or drink like most kids my age in this area” (man, 18-years-old).*

#### Misconceptions about TB causes

Participants also associated TB with dirt and an unhealthy lifestyle. One young man delayed linkage to TB care because he believed that he could not have TB because he lives in a clean environment. He also believed he was too young to have TB, he explained:

*“I don’t understand how I got TB, as you can see how clean this section is compared to other locations here. We have toilets and running water and they collect the bin [garbage] weekly. I thought you can get it [referring to TB] if you live in a dirty place with smelly water on the road. Look at me I’m young and fresh [smiling]” (man, 19-years-old).*

Most young people struggled to understand TB as a bacterial infection, even though they had a fairly good understanding about the risk factors and prevention measures when directly asked about them. A young woman associated TB with going out to public drinking spaces and with public health facilities. She claimed a possible place of infection was when visiting the PHC facility for reproductive health services.

*“I don’t understand how I got it because I barely go out to crowded drinking spaces where people smoke and might have TB and I use medical aid and go to private clinics. Maybe I got it the time I went for family planning at the local clinic” (woman, 27).*

#### Absence of TB symptoms

Some participants who delayed linking, indicated that they did not worry about TB because they felt relatively healthy. For example, a young man said that he was not concerned about his health even after being advised to get tested for TB. His response to testing for TB and checking for his results was also delayed, due to him feeling better after he was initially treated with antibiotics. As he was no longer felt sick, he did not bother to follow up:

*“If it wasn’t for my mother who kept pushing me to go to the clinic and those people from the clinic who kept coming back, I wouldn’t have gone back because I was not sick anymore” (man, 18-years-old).*

Similarly, a woman who had multiple previous TB episodes decided to ignore her diagnosis because she did not have any reason to think she had TB. She had experienced feelings of being sick in her previous TB episodes, but currently did not feel ill, and therefore questioned her TB diagnosis. She explained:

*“I have so many illnesses and taking medication for HIV and I didn’t go there for TB, I broke my leg. I know how TB feels like, when they told me about TB I didn’t really care. I already had TB 6 times remember, so what must I do?” (woman, 60years-old).*

The examples above illustrate that the absence of TB symptoms cause patients to question their TB diagnosis and thereby delay linkage to treatment.

### Motivation to reduce impact of illness on self and others

Motivation to link to care was related to minimising the physical impact of TB, being scared of severe illness that could result in hospitalization and not being able to continue with daily activities. Most participants who accessed TB care immediately after their diagnosis expressed wanting to get better and not to get to a point where other people would be able to see that they were ill. This included having clinical symptoms that could not be hidden or disguised from others. A young man explained:

*“Being sick is not a nice feeling and being seen by everyone on the streets and explaining yourself to people is even more stressful. The best way to avoid all this is to get help as soon as possible before things get out of hand” (man, 39-years-old)*.

Some participants used their family members’ past experiences as a motivation to link. One participant explained why he started treatment early and described his plan to follow the “proper” use of TB treatment and adhere to the healthcare worker’s instructions. He further explained that TB is curable if you adhere to your treatment and avoid drinking alcohol and smoking while on treatment:

*“These TB drugs work, I saw a lot of people getting better but you must use it properly, to help your body by taking a break at smoking and drinking too much beer so that your body can recover” (man, 35-years-old).*

The participant explained how he had observed a family member who died from TB and how this influenced his decision to seek TB care and follow the health providers’ instructions.

*“My uncle was killed by TB and I saw how he was because he was not taking his pills even after he discovered he had TB, so I was scared but I told myself that I must endure whatever is going to be done at me and do what I’m told” (man, 35-years-old).*

We found that most patients who linked without delay generally attributed this to both knowledge and a personal ‘drive’ to get better. A mother of two explained why she remained motivated which was mainly for the sake of her children:

*“My father had TB too and I know what TB does to you, my twin died from not taking TB treatment. He did not have any children but for me, I’m a single mother and I want to raise my kids. I am doing better with all the help from my aunt and friends” (woman, 43-years-old).*

Another participant explained that she understood that TB can be cured, as her partner recently completed TB treatment. However, her emphasis on seeking care and starting treatment immediately was to get well so that she could raise her children. She explained:

*“I am a mother of four boys and they are still young. I told you about the situation in this household and the lack of emotional and financial support from my husband, even though I did everything for him when he was the one sick. I really need to get well so that I can work and make money for my children” (woman, 45 years-old).*

## Discussion

This was an exploratory study with in-depth qualitative data from a purposive sample of three groups of TB patients linked to TB care almost immediately after diagnosis (without any intervention), or after an intervention (experienced a delay in linkage) or not linked to care, in a sub-district of Cape Town South Africa. We found that the majority of participants who had not yet been linked to care had received their diagnosis at the hospital and often had a previous TB disease (and treatment) episode–often with unpleasant experiences. Poor communication of the diagnosis by health services staff, and the failure to provide time and context for patients to understand that diagnosis relative to their past experiences or health beliefs, was a key challenge to linking. Participants who did not experience common TB symptoms, such as coughing and drenching night sweats, needed additional clarification of how they could still have TB. Participants highlighted poor support and previous unpleasant experiences engaging with TB services, which offered no opportunity for them to be supported incoming to terms with the implications of their TB diagnosis. This resulted in them losing confidence in the service and ultimately causing a delay in seeking care. Patients with previous unpleasant experiences, such as side effects from treatment or negative interactions with health services staff, especially needed assurances from health services that these would not be repeated.

Although several studies previously reported that there are significant delays in linkage to TB treatment, this is the first study that considers patient experiences after exposure to health service attempts to promote linkage (sms’, phone calls, and home visits). The breadth and depth of data collected over multiple interactions with the participants, not only summarised the participants’ reasons as presented by them but also located these relative to wider social contexts and illness histories.

As an exploratory study with a small sample of 5 participants from each of the 4 linkage categories (and many of the participants having been diagnosed in hospital not primary healthcare facilities), transferability is somewhat limited. However, a total sample size of 20 is typical of such studies, especially when data collection includes multiple interactions with each participant. Secondly, the study was conducted in one sub-district and therefore limited in terms of transferability to other areas with different underlying health system and epidemiological characteristics. Thirdly, our findings are descriptive but imposing theoretical framework or argument after the fact would not be scientifically sound. Future studies should consider this during planning and design. Lastly, we selected only adult TB patients and no interactions were made with family members’, children, and caregivers who are part of the care process from diagnosis to treatment. This could be explored in future studies.

Previous literature has shown that there are multiple health system challenges to linking TB patients to care. A study conducted in Zambia by Kaona, Tuba, Siziya, & Sikaona [17], showed that basic knowledge leads to a better understanding of the TB illness and has the power to influence people’s decisions positively. A study in Cape Town, South Africa by Furin et al., [18], showed that in the pre-treatment phase, nurses did not have adequate information for the patients to prepare and empower them with knowledge before embarking on the treatment journey. In 2018, an exploratory study by Moodley et al., [12] was conducted in the Free State Province, South Africa amongst patients and clinicians. This study showed how gaps in patient education and patient-centred approaches interfere with TB treatment. The results showed that patients felt that the TB messaging received was inadequate. Clinicians focused on treatment information and agreed that their TB medical management approach lacked the psycho-social dimension in treating a social disease of this scale. Another study conducted in the Free State Province of South Africa by Kigozi et al., [19], highlighted that most patients who delayed linking to care were patients who previously had TB as well as patients who had negative test results on the first sputum test and who were later confirmed with a positive TB diagnosis. These studies demonstrate the gaps in communication between health services and TB patients which contribute to delayed linkage to care. Our findings are in line with these previous studies, showing that the different diagnostic processes and all possible changes in the results were not clearly communicated with patients.

It is usually assumed that going through a similar medical experience for the second or third time should make it easier for the patient to understand. However, our results showed that even participants who had previously had TB, were uncertain about their diagnosis and had similar poor levels of understanding as those participants who were experiencing TB for the first time. Our study findings add to the body of literature that emphasizes the ongoing challenge in communication from healthcare workers to patients and how this significantly impacts linkage to TB care.

Too often, health system challenges have been positioned as the patients’ lack of understanding or knowledge and not acknowledging patients’ past experiences as part of the system problem. Various studies of TB patients with previous TB episodes showed that a person’s reaction to a TB diagnosis is highly influenced by previous treatment experiences and interpersonal relationships with health providers and affects decisions made in future Deshmukh et al., [20]; O’Donnell et al., [21]; van der Westhuizen and Dramowski [22]; Kielmann et al., [23]. A TB control analysis done in South Africa by Churchyard [24] showed that patients who previously had TB, could identify some of the common symptoms, but also delayed seeking care until the disease had progressed. The unfair assumption and expectation (often made by health workers) is that patients who have had TB previously understand the TB processes and should know what to do compared to those who are diagnosed for the first-time patients are problematic. Our findings support previous studies and show that most patients who previously had a TB illness might have an idea about the diagnosis but the past unpleasant treatment and interpersonal experiences with TB services are a barrier to future care. We suggest that the challenges highlighted in this study are about the gaps in the TB services and show communication gaps and lack of patient-centred care.

An approach in addressing health system challenges should be empowering and sensitive to people’s beliefs, traditions and cultural practices. This should be designed in a manner that educates people and opens a platform for different types of care and integrated services. A study by Abonyi et al., [25], looked at TB patients’ experiences of health and illness among the indigenous Canadians and showed that the absence of common TB symptoms such as coughing and drenching night sweats for some patients, caused delays in linking to TB care. As noted by O’Donnell et al., [21], “we should also consider the patient’s perspective and build on patient-provider relationships to enhance the humaneness of care through communication, shared decision-making and support for self-management”. Our study is consistent with this earlier work and that many of the same issues are experienced by patients in a sub-district in Cape Town, highlighting the lack of good interpersonal relations between health care providers and them. The health sector limits patients in exploring different sources of care and different explanations of illness. In addition, we have shown that patients have different beliefs and explanations of illness and the use of the biomedical only, in managing illness lessens chances of successfully managing TB. This could block an opportunity for people to have an open mind to discuss the biomedical explanations on causes of TB, new different treatment options and an open platform to discuss patients’ beliefs to get to a middle ground on the treatment plan and a way forward.

## Conclusion

Our findings suggest that people diagnosed with TB, especially in hospitals, require a specific plan to prepare and support them when transitioning from hospital to PHC facilities for linkage to care. Patients might be too ill to fully comprehend the TB diagnosis during hospitalization and having a limited understanding about TB, which can result in them confusing TB with other illnesses. The discussions revealed that a timely diagnosis of TB in a hospital or at PHC facilities did not always translate into immediate linkage to care for various reasons discussed above. Our study shows that there is a need for change in policy and in practice. The similarities in challenges experienced by patients diagnosed at PHC facility and in hospitals are evidence to communication issues. There should be new approaches on communication during the diagnostic, pre-treatment phases and before discharge for hospitalized patients between healthcare workers and TB patients. Community health workers and counsellors can drive an initiative to assess patient needs and assist in continued TB education and care that will suit individual needs.
